# The Steroid Saponin Protodioscin Modulates *Arabidopsis thaliana* Root Morphology Altering Auxin Homeostasis, Transport and Distribution

**DOI:** 10.3390/plants10081600

**Published:** 2021-08-04

**Authors:** Ana Luiza Santos Wagner, Fabrizio Araniti, Leonardo Bruno, Emy Luiza Ishii-Iwamoto, Maria Rosa Abenavoli

**Affiliations:** 1 Laboratory of Biological Oxidations, Department of Biochemistry, State University of Maringa, Maringa 87020900, Brazil; analuizawagner@hotmail.com; 2 Department of Agricultural and Environmental Sciences (DISAA), University of Milan, Via Celoria, 20133 Milano, Italy; fabrizio.araniti@unimi.it; 3 Department of Biology, Ecology and Soil Science, University of Calabria, Arcavacata di Rende (CS), 87036 Arcavacata di Rende, Italy; leonardo.bruno@unical.it; 4 Department of Agriculture, University of Reggio di Calabria, 89124 Reggio Calabria, Italy

**Keywords:** allelopathy, saponin, natural herbicide, specialized metabolites, phytotoxicity, auxin transport, root morphology

## Abstract

To date, synthetic herbicides are the main tools used for weed control, with consequent damage to both the environment and human health. In this respect, searching for new natural molecules and understanding their mode of action could represent an alternative strategy or support to traditional management methods for sustainable agriculture. Protodioscin is a natural molecule belonging to the class of steroid saponins, mainly produced by monocotyledons. In the present paper, protodioscin’s phytotoxic potential was assessed to identify its target and the potential mode of action in the model plant *Arabidopsis thaliana*. The results highlighted that the root system was the main target of protodioscin, which caused a high inhibitory effect on the primary root length (ED_50_ 50 μM) with morphological alteration, accompanied by a significant increase in the lateral root number and root hair density. Through a pharmacological and microscopic approach, it was underlined that this saponin modified both auxin distribution and transport, causing an auxin accumulation in the region of root maturation and an alteration of proteins responsible for the auxin efflux (PIN2). In conclusion, the saponin protodioscin can modulate the root system of *A. thaliana* by interfering with the auxin transport (PAT).

## 1. Introduction

Weeds are one of the major constraints for crop yield and quality due to the competition for water, light, and nutrients [1], showing high germination capacity, growth, and reproduction, even under adverse conditions [2]. To date, synthetic herbicide use is increasing since, due to their easy application and greater accessibility to farmers, they remain the most effective method for weed control [3]. However, their low biodegradability and persistence in agri-food products are the main causes of environmental pollution, food contamination, and human health injuries [4]. The intensive and indiscriminate use of herbicides has favoured the appearance of highly herbicide-resistant weed biotypes. There are currently 514 confirmed cases of resistant biotypes in 70 countries, among 262 species (152 dicotyledons and 110 monocotyledons), covering 23 of the 26 known herbicide sites of action [5].

Therefore, in past decades, the research has been focused on developing novel methods to limit or minimize these chemicals’ utilization [6]. Natural compounds, generally belonging to the secondary plant metabolism, offer potential advantages for seeking new molecules that are less harmful, easily degradable, structurally diverse, and target novel sites, compared to synthetic herbicides [7,8,9]. Therefore, they could be considered potential sources of new bioherbicides and/or as possible templates to develop novel agrochemicals [10,11,12]. Among these, coumarins, terpenoids, phenolic acids, and flavonoids, derived from plant residues or alive plant species with allelopathic properties and high biological activity, seem the most important source of bio-herbicides for weed control [7]. These compounds can directly or indirectly affect many physiological processes, such as cell division and elongation [13], nutrient uptake [14,15], photosynthesis [16,17,18], respiration [19] and hormone balance [20,21], by inducing different phenotypic responses [22,23].

The plant root system could be considered the first target of allelochemicals, which can modify its morphology and architecture in many plant species [20,24,25]. For example, the sesquiterpene farnesene strongly modified root morphology and anatomy, causing cellular damage, anisotropic growth, bold roots and a “left-handedness” phenotype in *Arabidopsis thaliana* (L.) Heynh. [26]. Coumarin strongly affected root morphology, causing a primary root reduction and increasing lateral root number and root hair length in *Arabidopsis* seedlings [27]. Similar results were observed with monoterpene citral [28], sesquiterpenoid nerolidol [20], alkaloid norharmane [25], and momilactone B [29]. All the authors suggested that these allelochemicals might alter the root system, affecting both the auxin signalling pathway and transport, and interfering with its spatio-temporal homeostasis.

Auxin plays a pivotal role in regulating root system growth and development by regulating cell division, differentiation and elongation, lateral root formation, gravitropic responses, and microtubule disorganization [30,31,32,33]. For example, the allelochemical benzoic acid inhibited primary root growth by increasing auxin accumulation in root tips and raising auxin biosynthesis and auxin polar transporters (PAT), AUX1 and PIN2, genes expression [24].

The involvement of specialized metabolites on plant growth and development and their influence on plant hormone homeostasis, especially auxin, has been widely documented [28,34]. Several studies demonstrated that quite a lot of secondary metabolites could modify root phenotype, altering auxin biosynthesis, signalling and/or transport. Among them, coumarin caused an alteration in root morphology, interacting with polar auxin transport (PAT) and modulating the influx or/and efflux proteins [27]. The coumarin derivative 4-methylumbelliferone was found to inhibit primary root growth and regulate lateral root formation by altering auxin redistribution [35]. By contrast, scopoletin, with a chemical structure similar to auxinic herbicide 2,4-D, fit into the auxin-binding site TIR1, exerting an auxin-like effect [36]. Moreover, the alkaloids narciclasine and norharmane significantly altered *Arabidopsis* growth, modulating auxin transport and inhibiting its biosynthesis [21,25,37,38].

Similar to auxin, reactive oxygen species (ROS) are often associated with root morphology alteration [39]. ROS accumulation can affect root growth [40], microtubule organization [26], and root hair formation [41]. Further, cross-talk between ROS and auxin to modulate root system elongation was already demonstrated [42,43].

Despite research efforts in secondary metabolites/auxin/ROS interaction, limited information is available concerning the saponin class.

The natural molecule protodioscin is a steroidal saponin commonly produced by monocotyledon species, such as different species belonging to the *Urochloa* and *Tribulus* genera [44,45]. In particular, *Urochloa ruziziensis (R. Germ. and C. M. Evrard)*, a forage crop widely used as a cover plant in a non-till system, can reduce weed emergence in the field [46] and greenhouse [47]. This effect is attributable to protodioscin, which was identified in the butanol extract of *U. ruziziensis* straw [48]. Nepomuceno et al. [49] reported that protodioscin isolated from *U. ruziziensis* also inhibits the growth of *Glycine max* (L.) Merr. seedlings in laboratory conditions. Despite reports on the phytotoxicity of protodioscin, its mechanisms of action have not yet been elucidated. A disturbance in mitochondrial respiratory activity and an oxidative stress condition was demonstrated in the primary roots of *Bidens pilosa* L. treated with protodioscin [50]. Recently, we demonstrated that the dicot *Ipomoea grandifolia* (Dammer) O’Donell and the monocot *Digitaria insularis* (L.) Fedde weed treated with protodioscin and butanolic extract of *U. ruziziensis* have drastic alterations in root morphology, including a reduction in the primary root length, the precocious appearance of lateral roots and reduction in root hairs [51]. The roots also exhibited features of advanced cell differentiation in the vascular cylinder. Although these morphogenic responses have suggested a disturbance in the auxin signalling, no direct evidence for this hypothesis has been examined [51].

In this respect, the present paper provides a deep insight into the protodioscin effects on *Arabidopsis* roots, a model species sensitive to secondary metabolites [52], to understand its mode of action through a physiological, biochemical, and pharmacological approach. The critical role played by the phytohormone auxin in the protodioscin-mediated effect is particularly investigated.

## 2. Results

### 2.1. Dose-Response Curves

**Figure 1:**
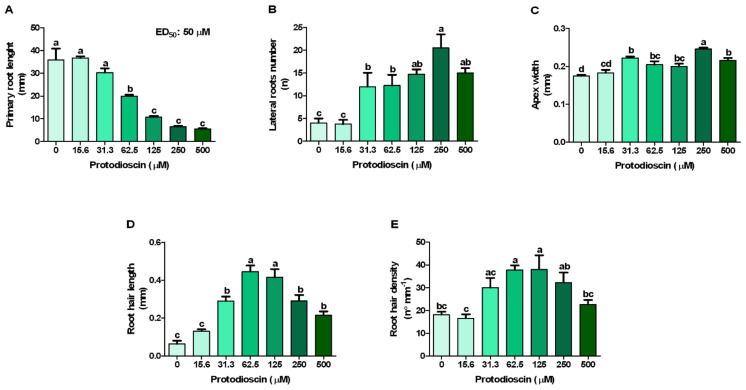
*Arabidopsis* roots morphology in response to the increasing doses of protodioscin: (**A**) primary root length; (**B**) lateral roots number; (**C**) apex width; (**D**) root hair length, and (**E**) root hair density. ED_50_: dose causing 50% reduction of primary root length. Different letters indicate significant differences observed among treatments at *p* ≤ 0.05 (SNK’s test). N = 4.

Although there is not a clear inhibitory effect at the lowest concentrations (15.6 and 31.3 μM), protodioscin significantly affected *A. thaliana* root growth, mainly at the concentration range of 62.5–500 μM, reducing the primary root length up to 85% (500 μM) (Figure 1A). The fitting of the raw data relating to this parameter, through non-linear regression, allowed to estimate the ED_50_ value equal to 50 μM (Figure 1A), the concentration adopted in the subsequent experiments.

At the lowest concentration (15.6 μM), protodioscin did not affect the number of the lateral roots, but it induced a strong stimulatory effect (2.5 times greater than the control) at 125 μM, reaching the maximum value at 250 μM (4 times greater than the control). Conversely, at the highest protodioscin concentration (500 μM), this positive effect was less marked (Figure 1B).

**Figure 2:**
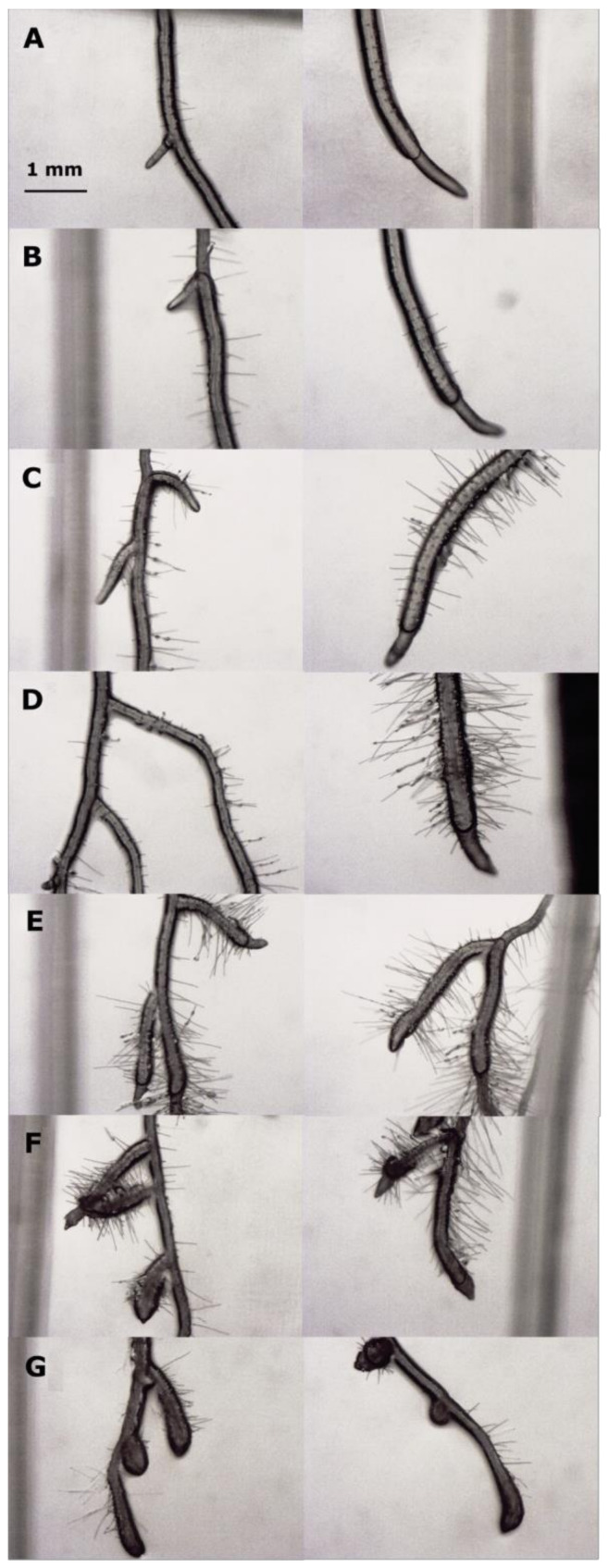
Stereoscopic microscopy images of *Arabidopsis* roots exposed to increasing doses of protodioscin: (**A**) control (0 μM); (**B**) 15.6 μM; (**C**) 31.3 μM; (**D**) 62.5 μM; (**E**) 125 μM; (**F**) 250 μM and (**G**) 500 μM. Scale bar 1 mm.

A similar trend was observed for apex width, for which protodioscin caused a thickening of the meristematic root area first detected at 31.3 μM (27% greater than the control) up to 250 μM (41%), after which its effect decreased again (23%, at 500 μM) (Figure 1C). Conversely, the root hair length and density were characterized by a gradual increase, reaching the maximum value at 62.5 and 125 μM, respectively, followed by a gradual reduction at the highest protodioscin concentration (Figure 1D,E, respectively). Root images confirmed the above results (Figure 2).

### 2.2. Protodioscin and Natural/Synthetic Auxin Interactions

**Figure 3:**
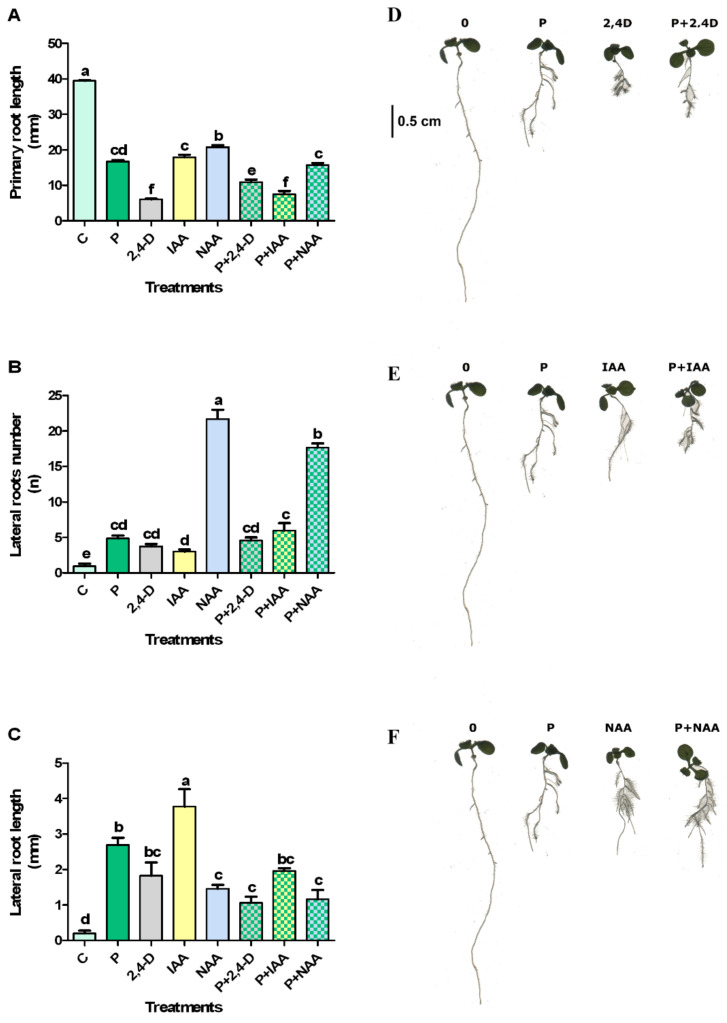
Primary root length (**A**,**D**), lateral roots number (**B**,**E**), and lateral root length (**C**,**F**) of *Arabidopsis* seedlings exposed to protodioscin (P) alone or in combination with auxins 2,4-D, IAA, and NAA (averaged data on the left side and representative image on the right side). Different letters indicate significant differences observed among treatments at *p* ≤ 0.05 (SNK’s test). N = 4.

Natural and synthetic auxins, alone or combined with 50 μM protodioscin, induced a significant primary root length reduction accompanied by an increase in number and length of lateral roots, compared to the control (Figure 3).

In particular, 2,4-D alone caused the highest inhibition on primary root length, but this negative effect was reduced in combination with protodioscin. By contrast, IAA and NAA combined with protodioscin increased their inhibitory effect, further reducing primary root growth (Figure 3A,D).

All the treatments significantly increased the number of lateral roots compared to the control (Figure 3B,E). In particular, NAA alone or in combination with protodioscin was the most effective treatment in stimulating the lateral root number. An additive effect was observed with IAA and protodioscin treatment. Contrastingly, no significant differences were observed between 2,4-D alone or in combination with protodioscin (Figure 3B,E).

Finally, protodioscin and IAA, alone or combined, were the most effective treatments in stimulating the lateral root length. Conversely, 2,4-D and NAA alone or in combination with protodioscin did not show any differences (Figure 3C). The images of *Arabidopsis* treated seedlings confirmed these results (Figure 3D,F).

### 2.3. Effects of Protodioscin and Auxin Inhibitors

**Figure 4:**
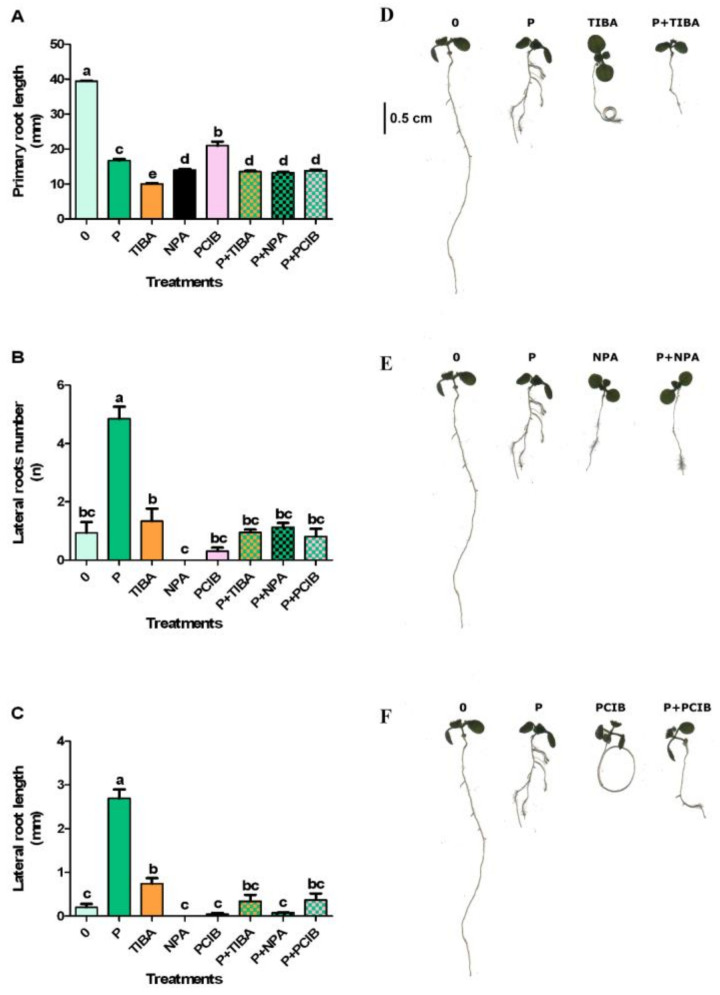
Primary root length (**A**,**D**), lateral roots number (**B**,**E**), and lateral root length (**C**,**F**) of *A. thaliana* seedlings treated with protodioscin (P), alone or in combination with auxin inhibitors TIBA, NPA, and PCIB (averaged data on the left side and representative image on the right side). Different letters indicate significant differences observed among treatments at *p* ≤ 0.05 (SNK’s test). N = 4.

Since the root morphological changes are generally related to an altered auxin distribution and/or accumulation, the effect of auxin transport inhibitors (TIBA and NPA) and auxin antagonists (PCIB), alone or in combination with protodioscin, was analyzed. The results showed that PCIB, TIBA and NPA, alone or in combination with protodioscin, strongly inhibited the primary root length (Figure 4A). Furthermore, in primary root, TIBA and PCIB treatments induced a circumnutating phenomenon typical of several transport and biosynthetic auxin inhibitors. Interestingly, this effect disappeared in combination with protodioscin, restoring the gravitropic root response (Figure 4D).

All the other treatments did not cause any significant effect, except for protodioscin, which significantly stimulated the lateral root number (Figure 4B,E).

Finally, the lateral root length was significantly stimulated only by protodioscin and, at a lower extent, by TIBA (Figure 4C,F).

### 2.4. Auxin Distribution through Auxin-Responsive Reporter pDR5::GFP and Its Relative Quantification

The dose-response curves and the pharmacological approach suggested that protodioscin caused strong root development and growth alterations, probably mediated by an alteration of auxin concentration and/or distribution. The *Arabidopsis* transgenic line for the auxin-responsive reporter *pDR5::GFP* was used to validate this hypothesis, and the auxin content was quantified.

**Figure 5:**
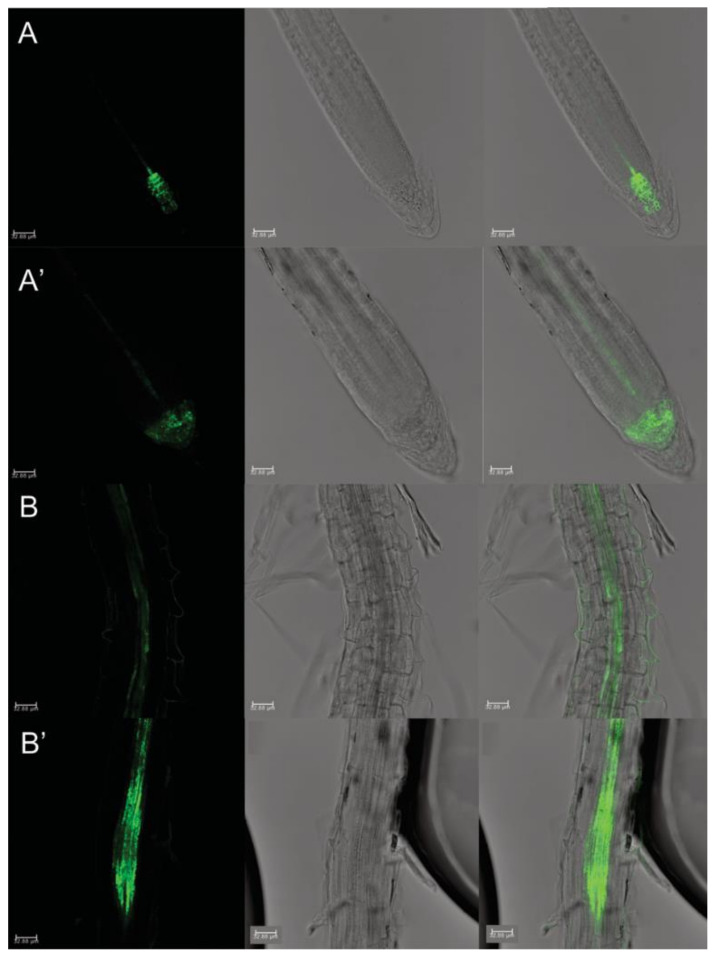
Primary root apex in seedlings of *Arabidopsis pDR5::GFP* transgenic line untreated (**A**) and treated with 50 μM protodioscin for 6 d (**A’**).Root maturation zone of *A. thaliana pDR5::GFP* transgenic line untreated (**B**) and treated with 50 μM protodioscin for 6 d (**B’**). Left side, GFP signal; center, transmission image; right side, merged image. Scale bars 32.88 μm. N = 4.

The *pDR5::GFP* transgenic line treated with protodioscin showed an evident impaired auxin distribution (Figure 5), displaying a maximum distribution in the quiescent center (QC) and initial columella cells, without extending to mature columella cells (Figure 5A’). Conversely, the control *pDR5::GFP* roots exhibited the typical auxin maximum distribution in the root tip (i.e., QC, initial and mature columella cells) (Figure 5A). Furthermore, treated *pDR5::GFP* roots displayed a stronger fluorescence signal locally in the elongation zone, specifically in xylem pole cells adjacent to the pericycle (Figure 5B), suggesting a potential auxin accumulation (Figure 5B’).

**Figure 6:**
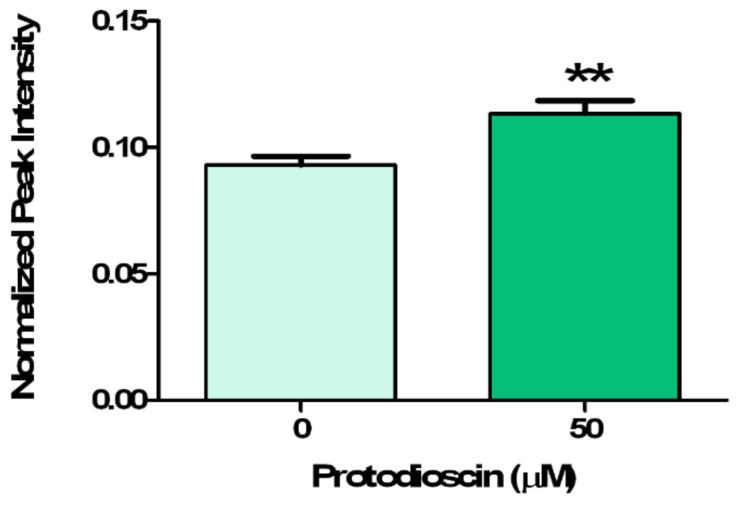
Relative quantification of IAA. Data are expressed as the average of the internal standard normalized intensity ± SE. Statistical significance of the data was evaluated through a *t*-test with *p* ≤ 0.05: * (*p* ≤ 0.05), ** (*p* ≤ 0.01), *** (*p* ≤ 0.001). N = 3.

This hypothesis was validated by the auxin quantification through GC-MS analysis, which pointed out a 21% accumulation of auxin higher than the control in mature root zone exposed to protodioscin (Figure 6).

### 2.5. Protodioscin Affected Auxin Gradient and Polar Transport

**Figure 7:**
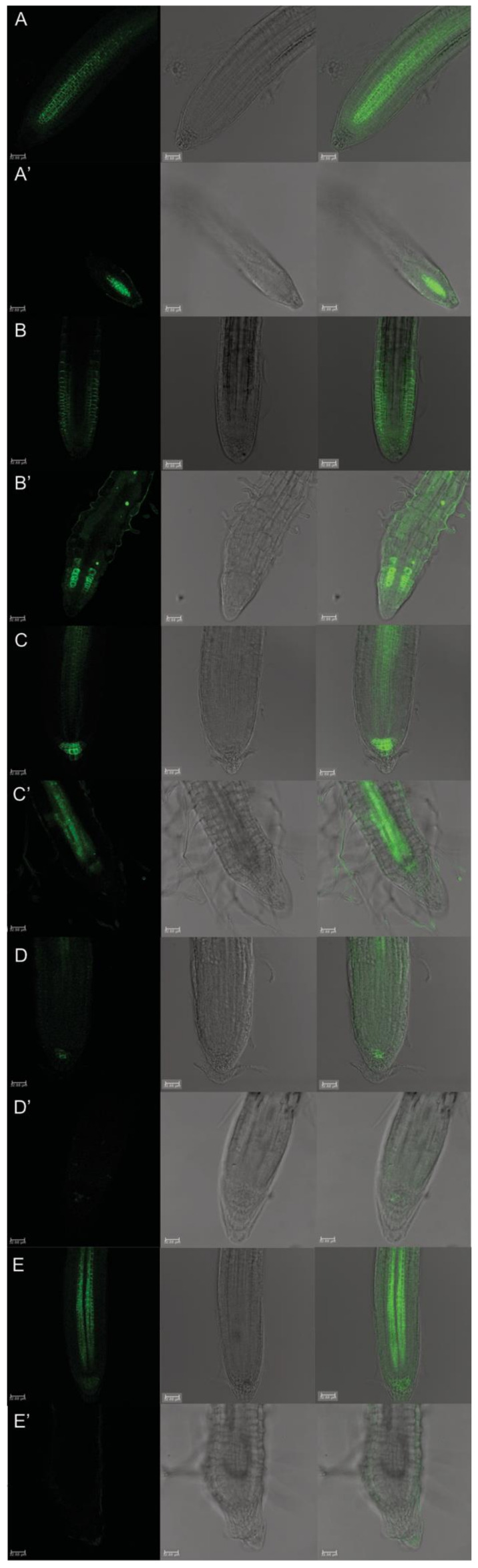
Primary root apex in seedlings of *Arabidopsis pPIN1::PIN1-GFP*, *pPIN2::PIN2-GFP*, *pPIN3::PIN3-GFP*, *pPIN4::PIN4-GFP*, *pPIN7::PIN7-GFP* transgenic lines untreated (**A**–**E**) and treated with 50 μM protodioscin for 6 d (**A′**–**E′**). Scale bars 32.88 μm. Left side, GFP signal; center, transmission image; right side, merged image. N = 4.

The PINs: PINs-GFP transgenic lines were adopted to detect the different auxin transport proteins involved in the polar auxin gradient in protodioscin-treated and -untreated root apexes (Figure 7).

The PIN1 resided at procambium and proendodermis cells, both in the meristem and distal elongation zone, in the untreated root apex (Figure 7A), while it was only revealed in a few cell layers of stele in the meristem zone in protodioscin-treated roots (Figure 7A’). The protodioscin treatment also affected the distribution pattern of PIN2 proteins, related to shootward/basipetal transport (Figure 7B,B’). In the control roots, the PIN2 proteins appeared at the apical side of the protodermis, at the lateral root cap cells, and mostly basally in the precortex cells, in the protodermis until the transition to the elongation zone (Figure 7B). Instead, protodioscin strongly altered the distribution of PIN2 proteins, whose signal was mainly localized in a bunch of proendodermal cells of the root meristem (Figure 7B’). The fluorescence signal was then diffused along the transition and elongation zone without a specific pattern, while no GFP signal was observed in the protodermis and precortex (Figure 7B’).

The localization of PIN3 protein was also altered by treatment (Figure 7C,C’). In the untreated roots, PIN3 proteins are localized in tiers two and three of columella cells, at the basal side of vascular cells, and on the lateral side of the pericycle cells of the elongation zone (Figure 7C). Otherwise, in the protodioscin treated roots, PIN3 proteins are localized in the procambium cells of the distal elongation zone only (Figure 7C’).

In the control, PIN4 and PIN7 proteins are mainly localized in provascular cells and all around the QC and surrounding cells, as well as in the provascular cells, meristem, and in the elongation zone, at the lateral and basal membranes (Figure 7D,E), respectively. They showed complete inhibition after protodioscin treatment in the root tips (Figure 7D’,E’).

### 2.6. In Situ Semi-Quantitative Determination of H_2_O_2_ and O_2_^−^

**Figure 8:**
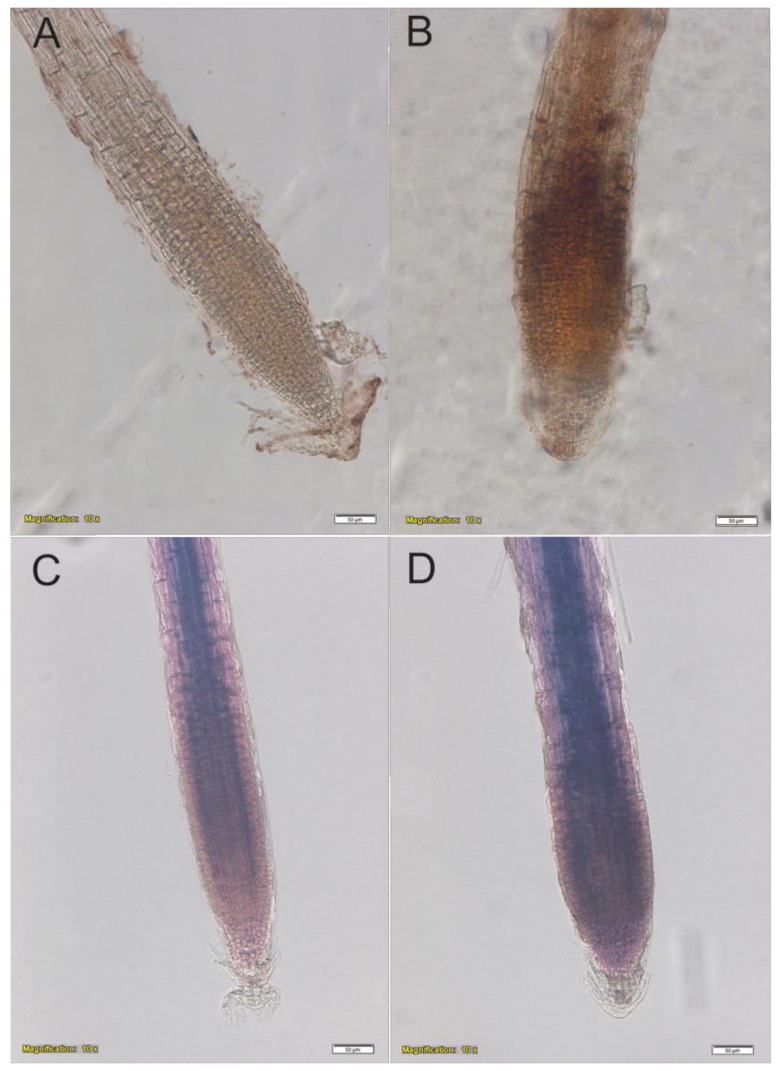
In situ hydrogen peroxide (dark brownish color) (**A**,**B**) and superoxide (dark blue color) (**C**,**D**) localization in roots of *A. thaliana* untreated (**A**,**C**) and treated with 50 μM protodioscin for 6 d (**B**,**D**). Image magnification 10×, scale bar 50 μm. N = 4.

Protodioscin (50 μM) caused a H_2_O_2_ increase in roots of *A. thaliana*, compared to the control (Figure 8A,B), without changing the O_2_^−^ production (Figure 8C,D).

## 3. Discussion

The in vitro assay revealed the inhibitory effects of protodioscin on *Arabidopsis* root growth, confirming the higher phytotoxic potential of this saponin [49,50,51], compared to other natural molecules [53]. The inhibition of primary root elongation was even observed at low concentrations, with an ED_50_ equal to 50 μM. A similar value was found in our previous study with the weed *I. grandifolia* (54 μg mL^−1^) and *D. insularis* (34 μg mL^−1^) [51]. An ED_50_ of 240 μM was found in *B. pilosa* by Mito et al., (2019). These values indicate that protodioscin has a higher inhibitory effect on weeds species when compared with the crop soybean. As reported by Nepomuceno et al. [49], protodisocin inhibits the root growth in soybean seedlings at a 680 μM concentration. The ED_50_ value (50 μM) was then used for all the subsequent experiments to identify its mode of action.

Protodioscin reduced primary root growth in a dose-dependent manner, causing strong root deformation at the highest concentrations. This reduction was also accompanied by an increase in lateral root number and root hair density, which hinted at a correlation with auxin homeostasis [54]. Furthermore, the saponin caused a thickening of the root apex, a phenomenon known as swelling, already observed with many natural compounds, such as coumarin [55,56], oryzalin [57], citral [58] and mainly attributable to an alteration of the cortical microtubules [59,60]. Similar results were also observed with the alkaloids narciclasine and norharmane [25,37], the sesquiterpene farnesene [26], which strongly affected microtubule organization and cellular ultrastructure of the meristematic apex in *Arabidopsis* roots, altering auxin transport and redistribution.

The root phenotype changes induced by protodioscin were associated with an increase in auxin content, underpinning that the protodioscin effect may be caused by the perturbation of auxin homeostasis. Similar effects were observed in *A. thaliana* seedlings exposed to phenylpropanoid 3,4-(methylenedioxy)cinnamic acid (MDCA) and benzoic acid [24,34].

Auxin plays an essential role in the root system by regulating cell division and differentiation in the meristematic and elongation zones. For example, it was proven that, under alkaline stress, the auxin accumulation in root tips negatively affected cell division in the meristem zone [61]. Recent studies pointed out that the auxin homeostasis perturbation led to allelochemical toxicity [61,62,63].

To provide an insight into the auxin–protodioscin interactions in *Arabidopsis* roots, natural and synthetic auxin, alone or in combination with the saponin, were added in the growth medium. The exogenous auxin applications did not ameliorate the inhibitory effects of protodioscin on primary root elongation; instead, it exacerbated the growth reduction, especially in combination with IAA or NAA, suggesting a negative additive interaction between them. Similar behaviour was observed with the lateral root number, which further increased in the presence of IAA or NAA and protodioscin. By contrast, the lateral root length was negatively affected by all the auxins co-supplied with protodioscin. Generally, in almost all the treatments, both the number and length of the lateral roots were still higher than the control.

Furthermore, the positive effect of saponin on the lateral root’s number and length disappeared when the auxin transport inhibitors (TIBA and NPA) or the auxin antagonist (PCIB) were added, suggesting that protodioscin was not able to overcome their inhibitory effect on lateral root formation. Interestingly, the addition of protodioscin partially restored the gravitropic root responses generally observed in seedlings treated with TIBA and PCIB [64] characterized by a lateral root lack and root coils, typical of mutants with altered auxin transport and distribution. All these results strongly suggested the auxin-like activity of protodioscin.

Likewise, the auxin-like effect of non-auxin probe naxillin (reduction in primary root growth and stimulation of lateral root number) was due to an auxin distribution and stimulation of the conversion of the auxin precursor indole-3-butyric acid into the active auxin indole-3-acetic acid in the root cap [65]. According to our results, plants treated with naxillin showed an induction of the synthetic auxin-responsive marker *pDR5::GUS* in the xylem pole cells adjacent to the pericycle of the basal meristem, demonstrating the impairment of auxin distribution and suggesting auxin accumulation in the root area involved in lateral root development [65].

To explore whether protodioscin induced auxin accumulation affecting PAT, we examined the role of the major auxin polar carriers, using the *PINs::GFP* transgenic lines. PAT is mediated by auxin influx carriers, AUXIN1/LIKE AUXIN1 (AUX1/LAX) and auxin efflux proteins, PIN-FORMED (PIN) [66,67]. In *Arabidopsis*, there are eight PIN proteins (PIN1–PIN8) that regulate auxin homeostasis [68]. Among them, PIN1 mediates acropetal auxin transport in the root stele; PIN2 is required for the basipetal flow of auxin through outer root cell layers; PIN4 is expressed around the columella cells and localized toward the QC, contributing to the auxin concentration in this tissue; PIN3 and PIN7 are responsible for the outward and inward lateral transport of auxin in the root cap and mature root zone, respectively [65,69]. Many natural compounds regulate root growth by affecting auxin homeostasis and PINs expression. Narciclasine inhibits auxin transport in *Arabidopsis* by mainly affecting the subcellular trafficking of PIN and AUX1 proteins through interfering with actin cytoskeletal organization [37]. The sesquiterpene farnesene induced auxin accumulation, mainly inhibiting PIN3 and PIN7 efflux carriers, which resulted in microtubule disorganization [70]. Recently, it was demonstrated that the allelochemical coumarin interferes with auxin polar transport, altering the microtubule cortical array organization and inducing, as in our experiments, root swelling and an increase in the lateral root number [60]. Furthermore, benzoic acid increased the auxin level in the root tips, associated with a higher expression of auxin biosynthesis and auxin polar transporter, AUX1 and PIN2 [24]. More recently, norharmane was shown to inhibit PIN2, PIN3 and PIN7 transport proteins, causing a significant inhibitory effect on the growth of *A. thaliana* seedlings [25].

Accordingly, protodioscin significantly interferes with all the PIN polar transporters, altering auxin distribution. The *PINs::GFP* imaged by confocal microscopy revealed a fall in the PIN4 and PIN7 GFP signal and an altered distribution in PIN1, PIN2 and PIN3 after protodioscin treatment. In further detail, PIN1 and PIN2 distribution were extremely reduced and localized only in the distal part of the root meristem, and PIN2 was only present on a few root meristem cells characterized by an abnormal shape. The alteration of the PIN1 proteins suggests a reduction in the downward movement of auxin (from the shoot to the root apex), although it was mediated by PIN3 proteins located in the stele region, which were less affected by the treatment. The modification induced by protodioscin of PIN2 could be responsible for a change in the root system plasticity as observed, especially under abiotic stress, such as aluminium and alkaline stress, which then inhibited primary root elongation by altering auxin distribution via disturbing the AUX1/PIN2-mediated auxin transport [60,71]. PIN3 was weakly expressed in the columella cells but highly accumulated in the stele. This distribution suggested a reduction in auxin in the QC and its accumulation in the elongation zone, as also observed using the auxin-inducible reporter *pDR5::GFP*. Furthermore, PIN4 and PIN7, which are involved in auxin lateral redistribution (PIN7) and downward distribution from the QC to the columella (PIN4) [65,67,72], respectively, were significantly altered at the QC and columella level, confirming an alteration of auxin distribution in the distal meristem and suggesting a potentially biased accumulation of auxin at the QC level.

Blilou et al. [66] reported that PIN1, PIN3 and PIN7 loss, combined with defective PIN2 induced a drastic reduction in primary root growth. These data might support the hypothesis that the alterations induced by protodioscin on PIN proteins might lead to the observed alterations in primary root development. Moreover, the accumulation in auxin observed in the xylem pole cells adjacent to the pericycle (*pDR5::GFP*) and the increase in fluorescence observed in PIN3 close to the elongation zone could suggest auxin accumulation (supported by the GC-MS) in the root pericycle, where lateral root initiation occurs. This could justify the protodioscin-induced increase in lateral root number observed in our experiments. Indeed, it is well known that auxin response maxTizioimally promotes a subset of xylem pole-associated pericycle cells to provide the competence to form lateral roots [73,74,75,76].

Root morphology changes, plant hormonal alterations, microtubule disorganization induced by phytotoxin generally caused higher ROS production [77,78]. ROS-induced oxidative damage is widely considered to be associated with allelopathic toxicity [17,79,80]. The gallic acid was reported to trigger high ROS levels in roots, leading to microtubule disruption and root architecture collapse [79]. Likewise, benzoic acid increased ROS levels in the meristematic, elongation, and mature root regions of *Arabidosis* [24]. In our study, plants treated with protodioscin in situ stained for H_2_O_2_ confirmed the increased accumulation in roots. This result is in agreement with our previous work showing an increased content of ROS in *I. grandifolia* and *D. insularis* roots treated with protodioscin [51]. Furthermore, this increase was also recently observed in roots exposed to nerolidol [20] and benzoic acid [24] and was accompanied by root morphological alterations.

## 4. Materials and Methods

### 4.1. Plant Material and Experimental Design

*A. thaliana*, ecotype Columbia (Col-0) seeds were sterilized and vernalized, as reported by Araniti et al. [70]. They were sown and then germinated on square Petri dishes (100 × 100 mm) containing agar (0.8% agar *w*/*v*) enriched with a mixture of micro- and macronutrients (Murashige-Skoog, Sigma-Aldrich SRL, Milan, Italy) and supplemented with 1% sucrose (*w*/*w*). The Petri dishes were then placed vertically in a growth chamber at 22 ± 1 °C and 120 μmol m^−2^ s^−1^ light intensity provided by a cold white fluorescent lamp (Polylux XL FT8, 55 W 8440, Barcelona, Spain), for a photoperiod of 8/16 h light/dark, and 55% relative humidity. After germination, five seedlings (4 days old) for each replicate were transferred to a single Petri dish containing the aforementioned medium supplemented with 0, 15.6, 31.3, 62.5, 125, 250, and 500 μM protodioscin (P) concentrations for 6 d and placed in the growth chamber under the same conditions described above. Protodioscin (Aktin chemicals Inc., Chengdu, China) was diluted in the medium by autoclaving. After 6 d of treatment, the primary root length was measured, and the average growth was calculated. These values allowed us to determine the ED_50_ value (dose causing 50% inhibition of the total response) used for all the subsequent experiments. The roots image was captured by scanning (STD 1600, Régent Instruments Inc., QC, Canada), and the primary and lateral root lengths (PRL and LRL, respectively) were measured using Image-Pro Plus v 6.0 software (Meia Cybernetics). The lateral root number was counted manually from the image (Abenavoli et al., 2008). The root hair length (RHL), density (RHD) (determined as the number of hairs in each of the apical segments (1 mm) of root observed), and apex width (AW) were determined by using stereoscopic microscopy (Olympus SZX9, Shinjuku, Tokyo, Japan) and Image-Pro Plus v 6.0 software (Meia Cybernetics, Rockville, USA).

### 4.2. Protodioscin and Auxins Interaction in Roots of A. thaliana

To determine the role of auxin on the protodioscin impact on the root system, a pharmacological approach was followed. In particular, the natural/synthetic auxins (2,4-dichlorophenoxyacetic acid (2,4-D), indole-3-acetic acid (IAA) and α-naphthalen acetic acid (NAA)), transport inhibitors (2,3,5-triiodobenzoic acid (TIBA) and N-1-naphthylphthalamic acid (NPA)) and an anti-auxin (p-chlorophenoxyisobutyric acid (PCIB)) were applied alone or in combination with protodioscin (50 μM) during *Arabidopsis* seedling growth. Seedlings were grown as previously described for 4 d and then transferred on an agarized medium enriched with mineral nutrients and the abovementioned compounds (Table 1).

### 4.3. Arabidopsis Transgenic Reporter Lines Bioassay

Seeds of *Arabidopsis* transgenic lines (background Columbia 0), and in particular, the synthetic reporter *pDR5::GFP*, and different auxin transport proteins *pPIN1::PIN1-GFP*, *pPIN2::PIN2-GFP*, *pPIN3::PIN3-GFP*, *pPIN4::PIN4-GFP*, and *pPIN7::PIN7-GFP*, were germinated and grown as previously reported. Five seedlings (4 d old) were then transferred to a single Petri dish containing the same medium previously described and enriched with 50 μM protodioscin for each treatment and replicate (N = 6). The transplanted seedlings were then placed in a growth chamber for 6 d and grown as previously described.

The *Arabidopsis* roots were then collected, fixed for 1 min in 4% (*w*/*v*) paraformaldehyde in 1X Phosphate Buffer Saline (PBS) (pH 7.0) and mounted in a 1:1 solution of glycerol: PBS (1X). Confocal images of median longitudinal sections were acquired using a Leica inverted TCS SP8 confocal scanning laser microscope, with a 40 × oil immersion objective. The detection of Green Fluorescent Protein (GFP) (excitation peak centered at about 488 nm, an emission peak wavelength of 509 nm) was performed by combining the settings indicated in the microscope’s sequential scanning facility. More than 20 seedlings were analyzed per treatment, and four independent experiments were carried out.

### 4.4. In Situ Semi-Quantitative Determination of H_2_O_2_ and O_2_^−^

Treated (P 50 μM) and untreated root tips (control) were cut, immediately immersed in distilled water, and vacuum infiltrated for 5 min with 0.65 mg mL^−1^ sodium azide solution (NaN_3_) in potassium phosphate buffer (pH 7.8) containing 0.1% (*w*/*v*) nitroblue tetrazolium (NBT), for O_2_^−^ detection.

For in situ H_2_O_2_ localization, treated and untreated root tips were transferred in acidified water (pH 3.8) containing 3,3′-diaminobenzidine (DAB) (1 mg mL^−1^) and infiltrated in vacuum conditions for 5 min.

After infiltration, the roots were incubated in darkness for 20 min in the same buffer and then illuminated until the stains appeared: reddish-brown or dark blue colour, for DAB or NBT, respectively [26]. The stained areas were determined by image analysis with the software Image ProPlus v.6.0 (Media Cybernetics Inc., Bethesda, MD, USA).

### 4.5. IAA Relative Quantification through GC-MS Analysis

Indole-3-acetic acid (IAA) quantification was carried out following the method proposed by Rawlinson et al. [81] with some modifications.

At the end of the protodioscin treatment, the *Arabidopsis* roots were snap-frozen in liquid nitrogen, powdered, and sorted in 2 mL vials, using 100 mg of plant material for each treatment and replicate. For sample normalization and IAA relative quantification, 20 μL of 3-indolepropionic acid (IPA) (20 mg∙mL^−1^) were added as an internal standard.

For IAA extraction, 200 μL of NaOH (1% *w*/*v*), 147 μL of methanol (MeOH), and 34 μL of pyridine were added, and the samples were vortexed for 40 s. IAA derivatization was achieved by adding to the extracted samples 20 μL of methyl chloroformate and vortexing for 30 s (the step was repeated twice).

To the derivatized extract, 400 μL of chloroform and 400 μL of NaHCO_3_ solution (50 mM stock) were added; the samples were vigorously vortexed for 20 s and then centrifuged at 14.000 rpm for 1 min to allow organic/inorganic layers separation. The organic lower phase was collected and dispensed into a new 2 mL centrifuge tube, and the aqueous residues were eliminated, using anhydrous Na_2_SO_4_. An aliquot (100 μL) of this organic phase was used for gas chromatography-mass spectrometry (GC-MS) analysis. A parallel experiment was carried out using pure IAA (Sigma Aldrich, 20149, Milano, Italy, Cat. No. I3750-25G-A) as an external standard for retention time (RT) assignment.

The GC-MS analysis was carried out using a Thermo Fisher gas chromatography apparatus (Trace 1310) equipped with a single quadrupole mass spectrometer (ISQ LT). The capillary column (MEGA -5MS, 30 m × 0.25 mm × 0.25 μm + 10 m of pre-column) (MEGA S.r.l., Legnano (MI), Italy) and the gas carrier was helium with a flow rate of 1 mL∙min^−1^. The injector and transfer lines were settled at 250 °C and 270 °C, respectively. A total of 3 μL of the sample was injected with a 35 psi pressure pulse, held for 1 min. The following temperature was programmed: isocratic for 1 min at 40 °C, from 40 °C to 320 °C with a rate of 20 °C × min, then isocratic for 2 min 320 °C. The ion source was settled at 200 °C, and the solvent delay was 4.5 min. Mass spectra were recorded in electronic impact (EI) mode at 70 eV, scanning at 50–400 m/z range. Then the MS was run in selected ion monitoring (SIM), using one quantifier ion (189 m/z) and two qualifiers (103 and 77 m/z) for IAA-Me ester identification. A mixture of alkanes was injected at the beginning of the experiment (C10-C40 all even) for retention index calculation. Finally, peak identification was carried out with the help of the commercial library (NIST 2011). The data were then expressed as a normalized (on an internal standard basis) peak intensity.

### 4.6. Statistical Analysis

All the experiments were carried out in a completely randomized design, with N = 4 for dose-response curves, N = 4 for pharmacological bioassay, and N = 3 for IAA quantification.

The dose-response curves data and pharmacological bioassay were expressed as mean ± standard errors (SE), and the data were analyzed using analysis of variance (ANOVA) with SNK’s test, post-hoc (*p* ≤ 0.05). Differences in auxin content were evaluated using the *t*-test (*p* ≤ 0.05).

The ED_50_ parameter was calculated, tightening the dose-response curve’s raw data through a non-linear regression log–logistic equation model. The equation was chosen from those that had the highest determination coefficient (r^2^) (best fit) (Software GraphPad Prism).

## 5. Conclusions

Our results provide an explanation for the molecular mechanisms underlying the effects of protodioscin on root growth. This molecule alters the hormonal balance, inducing auxin accumulation, and stimulates oxidative damage through the production of H_2_O_2_.

ROS production may alter the normal root growth, interfering with cell division and cytokinesis and consequently inducing root morphology alterations. The auxin accumulation could be the main reason for the increase in the number of lateral roots observed. Based on our results, we conclude that the saponin protodioscin is able to modulate the root system of *A. thaliana* by interfering with the auxin transport (PAT) and signalling.

## Figures and Tables

**Table 1:** Concentrations of natural/synthetic auxins, transport inhibitor, anti-auxin and/or protodioscin (P) used in the interaction assay.

Molecules	Concentrations μM
P	50
TIBA	15
NPA	5
PCIB	15
2,4-D	0.1
IAA	0.1
NAA	0.1
P + TIBA	50 + 15
P + NPA	50 + 5
P + PCIB	50 + 15
P + 2,4-D	50 + 0.1
P + IAA	50 + 0.1
P + NAA	50 + 0.1
